# Advances in research based on antibody-cell conjugation

**DOI:** 10.3389/fimmu.2023.1310130

**Published:** 2023-12-14

**Authors:** Xiaoxuan Ma, Jian Jiang, Xiaoye An, Wanting Zu, Chi Ma, Zhuo Zhang, Yaci Lu, Lijing Zhao, Lisheng Wang

**Affiliations:** ^1^Department of Rehabilitation, School of Nursing, Jilin University, Changchun, China; ^2^Department of Pharmacy, Tacheng People's Hospital, Tacheng City, Xinjiang Uygur Autonomous Region, China

**Keywords:** antibody-cell conjugation, cancer therapy, cancer cell therapy, monoclonal antibody, tumor targeting therapy

## Abstract

Antibody-cell conjugation (ACC) technology is a new research direction in medicine and biotechnology in recent years. The concept of ACC was proposed by Hsiao et al. and developed into a viable cell therapy technology, which refers to the cells with specific functions. Such as natural killer cells (NK cells), cytokine induced killer cells (CIK) and other immune cells and monoclonal antibodies through the linker together formed conjugate. ACC directly modifies specific antibodies on the cell surface through a simple and effective chemical coupling method to enable cells to have new functions. ACC has been developed for the treatment of various diseases, including cancers of the blood system and solid tumors. This paper reviews the current ACC construction methods, challenges and future development directions.

## Introduction

In recent years, cell therapy has shown some potential in cancer treatment. Cell therapy refers to the transplantation or input of normal or bioengineered human cells into the patient’s body, and the newly input cells can replace damaged cells (1), including stem cell therapy, and have stronger immune-killing function, so as to achieve the purpose of treating the disease immune cell therapy. The hottest cell therapy, chimeric antigen receptor T-cell (CAR-T) therapy, which was named the first cell-based gene therapy in the U.S., has been successfully applied to the treatment of acute lymphoblastic leukemia and non-Hodgkin’s lymphoma (2, 3). Studies have shown that CAR-T therapy can effectively improve the clinical outcome of hematologic malignancies; however, the therapeutic effect of CAR-T therapy on solid tumors is still limited because it is difficult to infiltrate the interior of large and dense solid tumors, and its targeted killing function is often inhibited by the tumor microenvironment (4). With the challenge of CAR, research interest has been focused on the cell surface, which is a highly dynamic interface with a complex three-dimensional network structure constructed from different types of proteins, lipids, and other carbohydrates on the surface of the cell membrane (5). Cell surface substances play key roles in intracellular signaling, intercellular and cell-environmental information transfer. Therefore, modification of the cell surface will help to study cell-cell interactions and the transduction of signals between cells and their downstreams, and this approach will also bring breakthroughs in cancer therapy (6).

**Figure 1:**
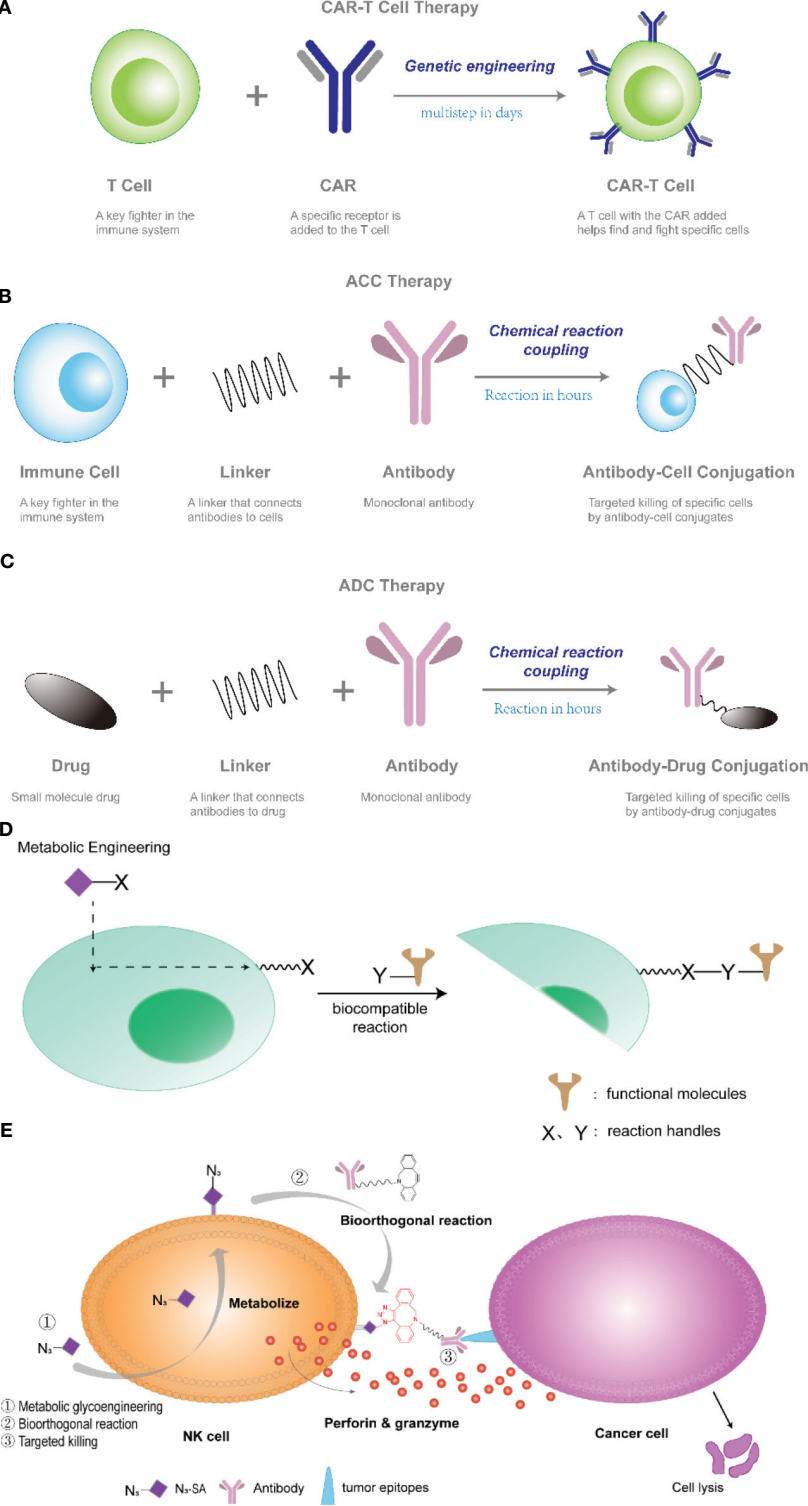
**(A–C)** Schematic diagram of CAR-T, ACC, and ADC technologies. **(D)** Cell surface engineering strategy based on one-step fucoidan glycosylation. Metabolic sugar engineering is used to mount a reaction handle (X) onto the cell surface that can react with a complementary handle (Y) on the target molecule. **(E)** NK cells modified with antibodies through metabolic sugar engineering and bioorthogonal reactions exhibited enhanced killing activity. NK cells, i.e. NK-92 cells, were metabolized with 9-azido N-acetylneuraminic acid methyl ester (N3-SA) to form NK-92-N3.DBCO-PEG4-NHS ester modification of a monoclonal antibody to form antibody-DBCO was facilitated by bioorthogonal reactions. azide-alkyne cycloaddition click reaction of functionalized NK cells with the antibody, attaching the antibody to NK-92-N3 cells.

Antibody-cell conjugation (ACC) technology, an emerging cell therapy technology in recent years, refers to the combination of immune cells with specific functions, such as natural killer cells (NK cells), cytokine-induced killer cells (CIK), and monoclonal antibodies through linkers to together to form a coupling (7, 8). The concept of ACC was proposed by Hsiao et al. (patent WO2015168656), and has until recently developed into a viable technology (9). ACC works similarly to CAR-T, serving to provide targeting for cellular therapies. The difference is that CAR-T technology requires genetic modification, which is time-consuming and labor-intensive; whereas ACC technology requires only a chemical reaction coupling, no genetic modification, and a much more controllable preparation time, as shown in **Figures 1A, B**. In addition, this powerful new approach to cell therapy may significantly enhance the ability of immune cells to recognize and kill tumors by unlocking multiple receptors signaling pathways.

Monoclonal antibodies (mAb) have been developed for the treatment of diseases such as cancer because of their ability to bind with high specificity, selectivity and affinity to antigens on the surface of specific cells. In 1900, Paul Ehrlich, a German physician, developed the concept of selectively delivering “magic bullets” to tumors through targeted agents. It was hypothesized that certain compounds could be directed to certain desired targets in the cell to treat disease (10). Due to many challenges in human antibody development, it was not until 1997 that the U.S. Food and Drug Administration (FDA) approved the first anticancer monoclonal antibody, rituximab, for the treatment of B-cell non-Hodgkin’s lymphoma (11). Until 2020, the FDA has approved about 30 mAb for the treatment of cancer, such as Avastin (12), trastuzumab (13), rituximab (14), cetuximab (15) and others, for the treatment of various solid tumors and hematologic cancers. mAb’s specificity also makes them ideally suited for the targeted delivery of drugs to cancerous cells, while avoiding normal tissues. As a result, antibody-drug conjugation (ADC) prepared by coupling antibodies to cytotoxic drugs have been developed in leaps and bounds in recent decades (16). ADC are targeting reagents that connect cytotoxic drugs to monoclonal antibodies via a junction, where the monoclonal antibody specifically recognizes cell surface antigens and delivers the toxic payload at the tumor site, thereby increasing chemotherapy efficacy and reducing systemic exposure and toxicity (17), as shown in **Figure 1C**. Since 2018, ADC has ushered in the second wave of development. At the same time, ACC is also receiving more and more attention.

The preparation principle of ACC is similar to that of ADC, which is to combine monoclonal antibodies with cells to form antibody-cell couplings, and to obtain ACC with target specificity by attaching antibodies, which can effectively reduce the damage to normal cells. In addition, ACC not only utilizes the targeting of antibodies to specifically recognize the surface antigen of the target site and play a therapeutic role, but also utilizes the natural activation signaling system of immune cells to recognize and kill the diseased cells and improve the therapeutic effect.

## Current status of ACC research

Research in ACC stems from revolutionary thinking about cancer therapy, and the development of ACC has been driven in large part by the development of recombinant DNA technology and monoclonal antibody engineering and modification, i.e., therapeutic live-cell delivery mediated by monoclonal antibodies to achieve efficient killing of tumor cells without causing significant damage to normal cells (18, 19). Monoclonal antibodies are antibodies produced by a single cell type against specific antigenic epitopes that are specific for a particular target. These antibodies can be used to specifically target and bind to specific types of cells, making them ideal for use in ACC. In addition, advances in gene therapy and gene editing have allowed researchers to create cells with specific functions, such as the ability to produce specific proteins or respond to specific stimuli; it is also possible to couple metabolic markers or chemical enzyme modifications introduced into different loci with the target cells through the use of genetic engineering (20). These advances have made it possible to create ACC with different functions that can be used to target and interact with specific cell types. Currently, several advances have been made in the study of ACC, and based on literature research, it has been found that researchers commonly use the following methods to construct antibody-cell conjugation:

First, coupling cells to monoclonal antibodies through metabolic sugar engineering and bioorthogonal reactions.

Metabolic sugar engineering is a widely used method of modifying cellular glycans with unnatural sugars. In this method, non-natural monosaccharide analogs are taken up by cells into the biosynthetic pathway, converted into activated phosphonucleotide donors, and doped into specific glycan structures by glycosyltransferases, as in **Figure 1D**. This technique cleverly utilizes the cellular metabolic sugar pathway to assemble sugar units with specific chemical groups into glycocalyxes on the surface of cell membranes, thus presenting reactive chemical groups on the cell membrane; then, through bioorthogonal reactions, polymers or nanomaterials containing phosphine or cyclooctyne can be mounted on the cell surface. Xianwu Wang et al. (21), by applying metabolic sugar engineering and bioorthogonal reactions that coupled cetuximab on the surface of natural killer (NK) cells to confer tumor-targeting ability to NK-92 cells. In this study, the authors first used metabolic sugar engineering to introduce an azide moiety (9-azido N-acetylneuraminic acid methyl ester) onto the surface of NK-92 cells to form NK-92-N3; second, the monoclonal antibody was modified with a DBCO-PEG4-NHS ester to form antibody-DBCO; and finally, the azide-alkyne click chemistry was facilitated by a bioorthogonal reaction coupling, attaching the antibody to NK cells, as shown in **Figure 1E**. The resulting NK92 cells (NK92-CET) had a high cytotoxic effect on cancer cells.

Metabolic glycoengineering provides a simple platform for conferring new chemical functions to glycan structures, and thus antibody-cell coupling enabled by metabolic glycoengineering is a promising strategy for enhancing anticancer immunotherapy.

Second, antibody-cell coupling is performed simply and efficiently by chemoenzymatic methods.

*In situ* glycan editing via glycosylases is a single-step approach to modify the cell surface glycocalyx. The most established example of its application is the *in vitro* fucosylation of MSCs and regulatory T cells using GDP-fucose (GF) and recombinant human α-1,3-fucosyltransferase (FucT) VI for the conversion of cell-surface α-2,3 sialic acid LacNAc residues to sialic acid Lewis X (22, 23). Currently, enzymatic glycoengineering of cell surfaces is not widely used for therapeutic interventions (24), mainly because current substrates for enzymatic transfer are limited to small synthetic molecules (MW < 5000) (25–27), whereas biopolymers with high therapeutic value (e.g., monoclonal antibodies) are not available.

**Figure 2:**
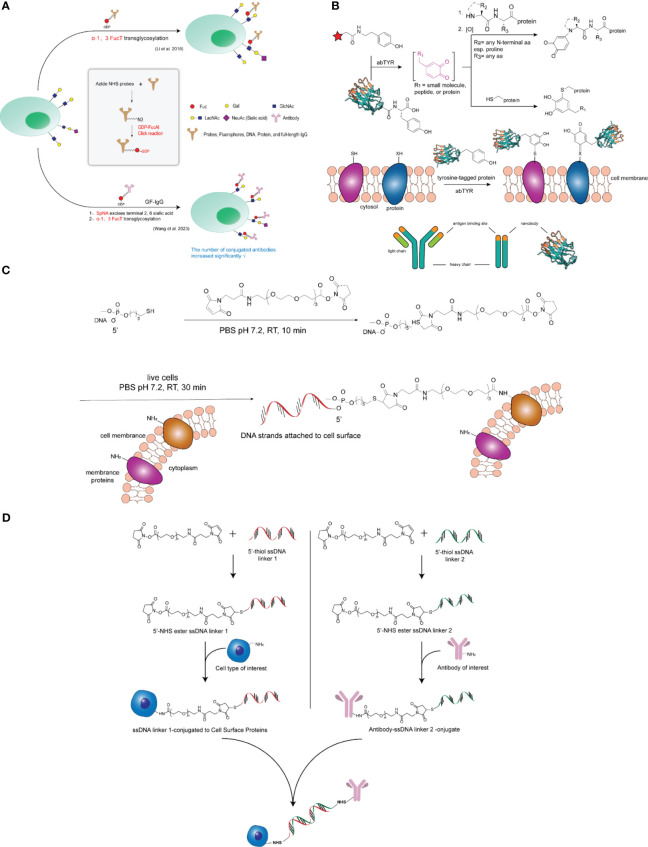
**(A)** Top: Workflow of FucT-catalyzed transfer of GF-IgG to the cell surface. Middle: synthesis of GDP-fucose coupled IgG (GF-IgG). Lower: Based on the construction of antibody-cell couplings using fucosyltransferase, the efficiency of α-1,3FucT-catalyzed fucose editing was further improved by pre-sialylation of the cells, thereby increasing the density of antibody coupling. **(B)** General strategy for modifying cell surfaces with nanobodies. Top: Tyrosinase catalyzes the oxidation of small-molecule phenols to highly reactive o-quinones, thereby modifying nucleophilic reagents present on proteins. Engineered tyrosine tags at the ends of proteins can also be oxidized by tyrosinase to produce site-specific o-quinones on proteins that react with protein-based nucleophilic reagents. Middle: Tyrosine-tagged nanobodies can be specifically oxidized by tyrosinase, causing these proteins to attach to cells. The resulting attachment creates a well-defined attachment site for mounting the nanobody on the cell surface while injecting new antigen-binding functionality into the target cell. Lower: Nanobodies are derived from low molecular weight (∼10-15 kDa) antigen-binding agents in the variable region of the camel antibody (PDB, ID: 3K1K). **(C)** Covalent attachment of ssDNA to the cell surface. Sulfhydrylated single-stranded DNA was first reacted with NHS-PEG-maleimide in PBS at room temperature to form an NHS-DNA coupling; the solution was then co-incubated with a suspension of live cells in PBS; the DNA strands were subsequently attached to the cells. **(D)** Antibodies were rapidly coupled to cell surface proteins. First, the NHS ester compound reacts with 5’ primed thiolated ssDNA to form ssDNA junction 1. ssDNA junction 1 will bind to exposed amide groups on cell surface proteins to form covalent bonds. Next, in a second reaction, the NHS ester compound reacts with the complementary 5’ vegetal thiolated ssDNA to form junction 2. junction 2 then reacts with the mAb. ssDNA junction 2 coupled to the mAb can be stored for up to 6 months prior to use. Finally, cells now affixed with ssDNA junction 1 are hybridized with the ssDNA junction 2-coupled mAb to form antibody-coupled cells.

However, Jie Li et al. (28) reported significant substrate tolerance of H. pylori 26695 α-1,3-FucT, an enzyme capable of transferring IgG antibodies coupled to GDP-fucose to common glycocalyxes (LacNAc and α-2,3-sialyl LacNAc) on the cell surfaces of living cells in minutes. In this study, the authors used H. pylori-derived α-1,3-fucosyltransferase (α-1,3-FucT) to construct the ACC. A one-step operation was used in this study to transfer biomolecules (e.g., IgG antibodies) onto the glycocalyx on the surface of living cells when the antibodies were coupled to the natural donor substrate of the fucosyltransferase, GDP-fucose, as shown in **Figure 2A**. This method does not require genetic modification, is rapid and biocompatible, and has little or no interference with endogenous cellular functions. In addition, the authors constructed two types of antibody-cell couplings (ACC) using a natural killer cell line (NK-92MI) and primary CD8+ T cells, and the modified cells exhibited specific tumor targeting and resistance to inhibitory signals generated by tumor cells, respectively.

In addition, based on Li Jie et al.’s study, Mingjing Wang et al. (29) added a step of pre-desalivation of cells to the construction of antibody-cell couplings using fucosyltransferase, which further improved the efficiency of α-1,3-FucT-catalyzed fucose editing, thereby increasing the number and density of antibody couplings, and provided a new method for high-density coupling of macromolecules on cells, as shown in **Figure 2A**. In this study, the authors found that trastuzumab-modified Jurkat cells had the ability to target HER2+ cells and enhanced binding affinity to HER2 cells compared with natural Jurkat cells.

In addition to fucosyltransferases, in recent years tyrosinase has been used for site-selective protein modification, which acts on free tyrosine amino acids and catalyzes their oxidation in melanin biosynthesis to highly reactive o-quinone intermediates, which can be used to modify the N-terminus of proteins as well as the free thiols present on the proteins (30, 31). Johnathan C. Maza et al. (32) reported a convenient enzymatic strategy for cell surface modification in a study of tyrosinase-mediated synthesis of nanobody-cell couplings. It has recently been shown that unique tyrosine residues introduced at the C-terminus of nanobodies can be selectively oxidized to reactive o-quinones using a tyrosinase isolated from Mushroom Agaricus bisporus (abTYR) and that the enzyme has been used for tyrosine-labeled single-chain variable fragments and site-specific modifications of full-length IgG (33, 34). These reactive intermediates are rapidly modified by nucleophilic thiol, amine, and imidazole residues present on the cell surface to produce novel nanobody-cell couplings with targeted antigen-binding capabilities. To demonstrate the applicability of this approach, the authors generated tyrosine-labeled nanobodies that are small antigen-binding agents derived from the variable region of camel immunoglobulin (35). The results show that the abTYR site specifically oxidizes the introduced tyrosine label and mediates the attachment of the nanobodies to the surface of natural killer (NK) cells while retaining their antigen-binding capacity, as shown in **Figure 2B**. In addition, the authors extended this approach to the synthesis of nanobody-NK cell couplings for targeted immunotherapy, and the resulting nanobody-cell couplings possessed novel antigen-binding functions. Thus, this strategy also provides a simple and straightforward alternative to genetic engineering synthesis for cell-based immunotherapy applications.

In summary, antibody-cell conjugation can be performed simply and efficiently by chemoenzymatic method, which provides a new idea for the research and development of cell therapy and has a broad potential application value.

Third, NHS-DNA couplings were utilized to directly modify the cell surface.

Sonny C. Hsiao et al. reported a method of hybridizing synthetic DNA strands attached to the cytoplasmic membrane of living cells in a previous study (36). The method simply involves first feeding the cells with an azide-containing mannose derivative for 1-3 days, followed by the sugar being metabolized by being doped into a cell surface glycan containing sialic acid, and finally targeting the DNA to the azide function by Staudinger ligation (37). This method, although effective, is still best suited for culturing mammalian cell lines because it requires several days of exposure to install a sufficient number of azide moieties. Next, the authors, in an effort to extend the versatility of this DNA-based adhesion method, developed an effective and improved method capable of mounting DNA strands directly onto virtually any cell surface, which directly modifies the cell surface with DNA strands using NHS-DNA couplers (9). The general strategy for direct modification of cell surfaces with DNA strands is to target amino groups present on membrane proteins to couple single-stranded DNA to the cell surface, as shown in **Figure 2C**. This approach enables the capture of many novel cells at specific surface locations in a sequence-dependent manner. This strategy allows the creation of complex networks of living cells by self-assembly.

Matthew J. Frank et al. (8), in their study of a novel antibody-cell coupling method for the enhancement and characterization of cytokine-induced killer (CIK) cells, drew on NHS-DNA coupling to illustrate that the antitumor activity of CIK cells can be enhanced by a novel method that uses a three-step approach to attach tumor-targeting antibodies directly and rapidly to cells surface proteins, as shown in **Figure 2D**. First, single-stranded DNA (ssDNA) is coupled to a therapeutic mAb; second, complementary ssDNA is coupled to the surface proteins of the CIK cells; and finally, the modified antibody is attached to the modified cells by hybridization of the complementary DNA strands. Because the CIK cell product is a heterogeneous population of lymphocytes containing a high percentage of CD3 + CD56 + cells with a natural killer T cell-like phenotype, CIK cells are an emerging cell therapy option for the treatment of cancer. It was found that CIK cells after antibody coupling had better and more efficient cytotoxicity compared to conventional CIK cells using *in vitro* killing assays. In addition, this study demonstrated the potential to selectively analyze the cell surface proteome of CIK cells using this antibody-coupled cell approach and found that they are enriched in proteins involved in immune response and cell activation.

In addition, Haokang Li et al. (7) also utilized NHS-DNA coupling to couple trastuzumab to NK cells, modifying the cell surface and the antibody with single-stranded DNA (ssDNA), respectively; the modified cells and the antibody were further coupled via complementary strands, as shown in **Figure 2D**. In this study, the authors constructed a new endogenous CD16-expressing oNK cell line (oNK) with antibody-dependent cytotoxicity (ADCC) as well as one that would preferentially express NK-activated receptors and low-level inhibitory receptors (38). The authors applied the ACC platform to couple trastuzumab on oNK cells and demonstrated its *in vitro* and *in vivo* efficacy against HER2-expressing cancer cells. The final product, cryopreserved and irradiated trastuzumab-NK cell coupling (ACE1702), was obtained and inhibited HER2-expressing cancer cells *in vitro* and *in vivo* and showed no tumorigenicity. This study provides a rationale for promoting this coupled drug as a safe and effective off-the-shelf cell therapy for HER2-expressing cancers.

## ACC challenges and future prospects

Antibody-cell conjugation technologies have a wide range of applications in biomedical research and therapeutic areas. One such company, Acepodia, is a clinical-stage biotech company utilizing its unique ACC platform technology to develop first-in-class cellular therapies to address gaps in cancer treatment. Using ACC technology, antibodies targeting tumors are linked to their proprietary immune cells to create new Antibody Cell Effect (ACE) therapies that enhance the strength of binding to tumors with low expression of antigens. One of the company’s fastest-progressing product candidates in development, ACE1702, which uses the ACC platform to modify the cell surface with single-stranded DNA (ssDNA), is an antibody-NK cell-coupled drug candidate that is currently in Phase I and is being developed for the treatment of HER2-expressing solid tumors. In addition, the Company has five other preclinical stage product candidates, ACE1975, ACE2016, ACE1831, ACE1708, and ACE2023.However, there are challenges associated with the ACC technology, and the following are some of the challenges that may exist:

First, selectivity and specificity: First, ensuring that antibodies bind to target cells with a high degree of selectivity and specificity is an important challenge. Antibodies need to be able to distinguish between target cells and other cells to avoid adverse effects or inadvertent injury to normal cells. Second, delivering the antibody to the target cell location is also a challenge, especially when it is necessary to cross biological barriers or access hard-to-reach tissues. Therefore, monoclonal antibodies for coupling need to be as selective and specific as possible.

Second, production and purification: there have been many genetic and chemical approaches to modify antibodies to mitigate side effects while maintaining therapeutic efficacy. Genetic manipulation of antibodies allows for the production of fragmented antibodies that have been shown to be therapeutically acceptable with an enhanced safety profile (39). However, the high-end technology involved in the process raises the cost of the final product, and large-scale production and efficient purification of specific antibodies can be a challenge, especially when customized antibodies are required. The production process needs to be reliable, efficient, and able to maintain the biological activity of the antibody (40).

Third, stability: the stability of antibody-cell couplings can be affected by the *in vivo* environment, which may lead to inactivation or breakdown of the antibody *in vivo*, thus affecting therapeutic efficacy.

Fourth, dose and frequency: determining the appropriate antibody dose and frequency of administration for optimal therapeutic efficacy may require further study.

Fifth, resistance: Long-term antibody therapy may lead to the development of resistance in the target cells, which may diminish the therapeutic effect.

Sixth, therapeutic persistence: it has been shown that ACE1702 administered intraperitoneally is detectable in the peritoneal region for 7 days, and intravenously administered ACE1702 lasts for 14 days (7). Therefore, it is also a challenge to ensure that the therapeutic effects of antibody-cell coupled drugs are sustained over a period of time rather than transient effects.

Seventh, safety: Antibody-cell coupled drugs may trigger adverse reactions, such as cytotoxicity or inflammatory responses, and require detailed safety assessments. Over the past decade, immune checkpoint blockade (ICB) therapies have made remarkable progress in cancer treatment. Despite the high therapeutic efficacy of ICB, only 20-30% of patients have been shown to benefit from monotherapy and the majority of patients do not respond. Therefore, the trend is to increase the dose of ICB therapy or pair ICB with other therapies to overcome the low response rate of ICB. Since normal cells also express immune checkpoints and share antigens with tumors, ICB inevitably causes inflammatory side effects in normal tissues, which are known as immune-related adverse events (irAEs) (41). These irAEs resemble autoimmune reactions and typically affect the skin, gastrointestinal tract, lungs, endocrine and musculoskeletal systems. These toxic effects remain a major challenge in clinical care and a barrier to developing more aggressive combinations.

Finally, clinical translation: the process of translating from laboratory research to clinical application may require overcoming challenges at multiple levels, including regulatory, manufacturing, and clinical trials. However, we believe that the challenges faced by ACC technology will be overcome as technology develops and research progresses. In response to these challenges, we researchers still need to continue our efforts to improve the antibody-cell coupling technology and realize its wide application in therapeutic and diagnostic fields.

ACC technology is an excellent and rapidly evolving area of tumor-targeted therapy today, combining the ability of monoclonal antibodies to specifically target tumors with potent tumor cell killing activity. Although the currently available ACC already offer great advantages, there are still areas that need to be optimized. In order to allow more cancer patients to benefit from antibody-cell coupled drugs, future research directions may be:

First, further clarification of the anti-cancer mechanism and organ damage mechanism, enhancement of targeting, and elimination of toxic side effects caused by off-target effects.

Second, target-specific studies can be performed on ACC for bystander effects and other characterizations, while identifying predictive biomarkers and providing mechanistic insights to support clinical decision-making.

Third, the prognosis of patients with tumors may improve with the treatment of ACC drugs; however, cancer cells can develop resistance and patients can ultimately deteriorate as a result. There are usually two mechanisms of drug resistance: primary resistance, which is often caused by mutations in target genes, and acquired resistance, which is usually categorized into dependent and non-dependent resistance. Further studies should be conducted to elucidate the possible mechanisms of resistance to ACC drugs in relevant malignant tumors, and appropriate countermeasures should be taken to solve the drug resistance problem so that more tumor patients can be benefited.

Finally, researchers should also conduct in-depth studies on the drug delivery and intracellular release mechanisms of ACC, including the endocytosis pathway mediated by endonuclease, the endoplasmic reticulum pathway mediated by acidic endoplasmic reticulum, and the lysosomal pathway mediated by lysosomes, in order to realize more precise intracellular drug release as much as possible in order to improve the therapeutic effect.

In conclusion, antibody-cell couplings, as an emerging therapeutic approach, have shown extensive potential in the treatment of a variety of diseases and are continuously being researched and developed. Currently, there are researchers working to improve the therapeutic efficacy and safety of ACC and exploring their applications in more fields.

## Author contributions

XM: Writing – original draft, Writing – review & editing. JJ: Writing – review & editing, Writing – original draft. WZ: Writing – review & editing, Supervision. CM: Writing – review & editing. ZZ: Writing – review & editing. YL: Writing – review & editing. LZ: Formal analysis, Supervision, Writing – review & editing. LW: Writing – original draft, Writing – review & editing. XA: Writing – review & editing.
